# Sorbitol signaling: Linker histone MdH1.1 modulates malic acid buildup in apple

**DOI:** 10.1093/plcell/koae332

**Published:** 2024-12-18

**Authors:** Maneesh Lingwan, Arpita Yadav

**Affiliations:** Assistant Features Editor, Plant Physiology, American Society of Plant Biologists; Donald Danforth Plant Science Center, St. Louis, MO 63132, USA; Assistant Features Editor, The Plant Cell, American Society of Plant Biologists; Department of Biology, The Pennsylvania State University, University Park, PA 16802, USA

The taste and flavor of apple (*Malus domestica*) and other fleshy fruits depends on the balance of soluble sugars and organic acids. Malate, the primary organic acid in apple, is a tricarboxylic acid cycle intermediary, mitochondrial respiratory substrate, cytosolic pH regulator, stomatal function supporter, and carbon energy storage molecule. A tonoplast malate transporter, aluminum-activated malate transporter 9 (ALMT9/Ma1), underlies the malic acid (*Ma*) locus in apple and is largely responsible for the buildup of malic acid in the vacuole (Li et al. 2020a, 2024). The source-sink ratio in apple trees (proportion of photosynthetically active leaves to developing fruits) also affects fruit acidity as well as sugar levels and fruit size, but the underlying genetic mechanism linking fruit acidity to sugars remains unknown.

Sorbitol is the predominant photosynthate and transport sugar (alcohol) in apple and other tree fruits in the Rosaceae family. Aldose-6-phosphate reductase (A6PR) converts glucose-6 phosphate to sorbitol 6-phosphate (S6P), which is dephosphorylated into sorbitol by S6P phosphatase. Previous research showed that antisense suppression of *A6PR* lowered fruit titratable acidity at harvest in addition to altering carbohydrate metabolism in both leaves and fruit (Cheng et al. 2005; Teo et al. 2006). In new work, **Da-Gang Hu and colleagues (Hu et al. 2024)** characterize the developmental profiles of apple sugars and malate in response to *A6PR* suppression and show that antisense *A6PR* fruits have lower malate levels throughout fruit development, corresponding to their lower sorbitol levels. RNA-seq analysis identified differentially expressed genes involved in malate metabolism and transport. These included the transcripts of genes encoding MdALMT9 (Ma1), P-type ATPase MdPH5, MYB transcription factor MdMYB73, and cold-induced helix-loop-helix transcription factor MdCIbHLH1, which were significantly decreased in transgenic fruit lines across all developmental stages compared with the wild-type control. The authors also found that a linker histone (H1), MdH1.1, was strongly coexpressed with MdMYB73, a previously characterized transcriptional activator for *Ma1*, in response to sorbitol. These findings suggested that lower malate buildup in the antisense lines is linked to the decreased expression of these potential candidate genes.

Sorbitol is not only a key substrate for carbohydrate metabolism but also acts as a signal regulating gene expression for its own metabolism, flower development, pollen tube growth, and fungal resistance in apple (Meng et al. 2018a, 2018b; Li et al. 2020b; Meng et al. 2023). The work of Hu et al. expands the signaling role of sorbitol to malic acid accumulation by linking sorbitol to the expression of a linker histone gene *MdH1.1* (Fig.). Linker histones are fine-scale architects for chromatin structures and interact with other proteins for epigenetic regulation of gene expression (Rutowicz et al. 2019). Hu et al. (2024) show that, in addition to being an architectural protein for chromatin structures, MdH.1.1 acts as a transcription factor, directly regulating gene expression in apple. MdH1.1 activates the expression of *MdMYB73*, *MdCIbHLH1*, and *MdPH5* by directly binding to specific cis-regulatory elements in their promoters. In return, MdMYB73 binds to the promoter of *MdH1.1* to enhance its transcription. The expression of *MdALMT9* (*Ma1*) is regulated by this MdH1.1-MdMYB73 positive feedback loop in response to sorbitol (Fig.). RNAi inhibition of *MdH1.1* or *MdMYB73* considerably reduced sorbitol-induced malate accumulation in fruit and transgenic calli, in contrast to their overexpression, which promoted *Ma1* expression and malate accumulation in transgenic calli.

**Figure.**
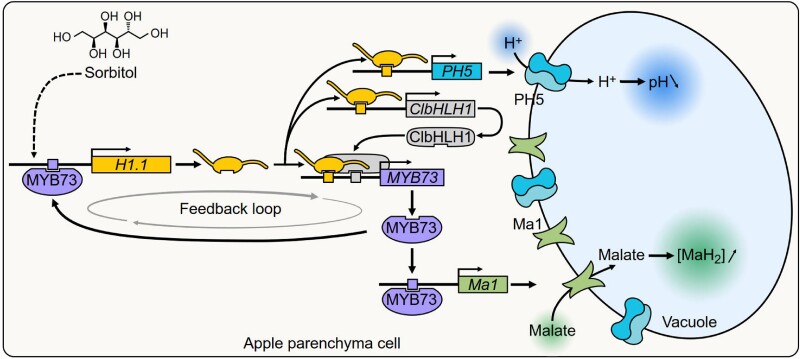
The model demonstrates how sorbitol modulates malate accumulation in apples through the linker histone MdH1.1. MdH1.1 functions as a transcription factor that responds to sorbitol and activates the expression of the transcription factor MdMYB73. Subsequently, MdMYB73 enhances the expression of MdH1.1, further strengthening the sorbitol signaling pathway. This positive feedback mechanism promotes the expression of *Ma1* encoding the aluminum-activated malate transporter MdALMT9 (Ma1), facilitating the transport of malate into the vacuole across the tonoplast. This regulatory loop also connects Ma1-mediated malate transport with vacuole acidification via P-ATPase MdPH5 and the interaction between MdCIbHLH1 and MdMYB73, along with the transcriptional activation of MdMYB73. Reprinted from Hu et al. (2024), Figure 11.

The work of Hu et al. (2024) elucidates the role of sugar signaling in modulating vacuolar malate transport in plants via the linker histone MdH1.1. It uncovers a new function of linker histones in regulating gene expression, revealing another facet of this group of multifunctional proteins that are essential to plants and other eukaryotes. On the sugar signaling side, examining the impact of sorbitol on malate accumulation in plant species with trace amounts of sorbitol may reveal a larger role and evolutionary importance of sorbitol signaling.

## Data Availability

No new data were generated or analysed in support of this research.

## Dive Curated Terms

The following phenotypic, genotypic, and functional terms are of significance to the work described in this paper:

MdMYB73 Gramene: MD08G1107400
MdMYB73 Araport: MD08G1107400

MdALMT9 Gramene: MD16G1045200
MdALMT9 Araport: MD16G1045200

MdCIbHLH1 Gramene: MD14G1148600
MdCIbHLH1 Araport: MD14G1148600

MdPH5 Gramene: MD17G1155800
MdPH5 Araport: MD17G1155800
